# Supplementary material for: Splice-Junction-Based Mapping of Alternative Isoforms in the Human Proteome
Source: Cell Rep. Author manuscript; Available in PMC 2020 Jan 15. (PMC6961840; doi:10.1016/j.celrep.2019.11.026)
Supplement: 2 [file NIHMS1546469-supplement-2.zip › DF1/PXD009737/TestisGluC/TestisGluC_21_PHC1_KICAKINVLKEQSGHHIGRPAHQPIPE.pdf]

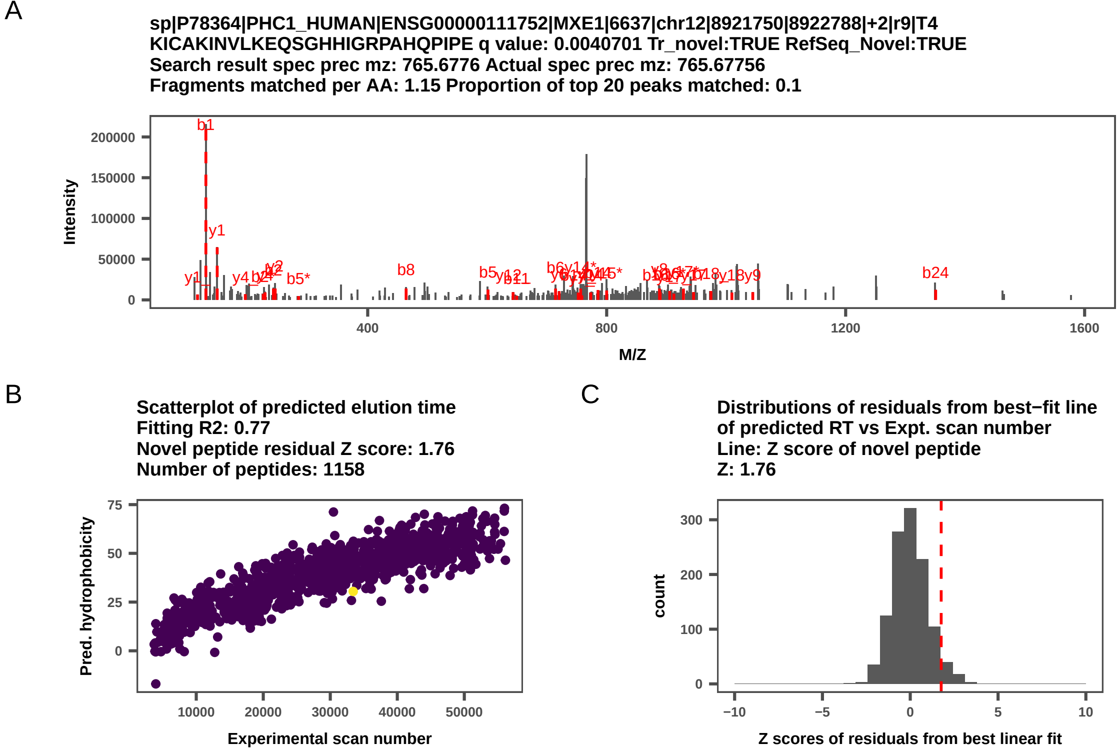
A
sp|P78364|PHC1_HUMAN|ENSG00000111752|MXE1|6637|chr12|8921750|8922788|+2|r9|T4
KICAKINVLKEQSGHHIGRPAHQPIPE q value: 0.0040701 Tr_novel:TRUE RefSeq_Novel:TRUE
Search result spec prec mz: 765.6776 Actual spec prec mz: 765.67756
Fragments matched per AA: 1.15 Proportion of top 20 peaks matched: 0.1
Intensity
200000
150000
100000
50000
0
b1
y1
b5*
b8
b24
400
800
1200
1600
M/Z
B
Scatterplot of predicted elution time
Fitting R2: 0.77
Novel peptide residual Z score: 1.76
Number of peptides: 1158
Pred. hydrophobicity
75
50
25
0
10000
20000
30000
40000
50000
Experimental scan number
C
Distributions of residuals from best−fit line
of predicted RT vs Expt. scan number
Line: Z score of novel peptide
Z: 1.76
count
300
200
100
0
−10
−5
0
5
10
Z scores of residuals from best linear fit
